# FM1-43 is a permeant blocker of mechanosensitive ion channels in sensory neurons and inhibits behavioural responses to mechanical stimuli

**DOI:** 10.1186/1744-8069-3-1

**Published:** 2007-01-06

**Authors:** Liam J Drew, John N Wood

**Affiliations:** 1 Dept. of Biology, UCL, Gower Street, London, WC1E 6BT, UK; 2 Dept. of Physiology and Cellular Biophysics, Columbia University, 630, W168th St, New York, NY10032, USA

## Abstract

The molecular identity and pharmacological properties of mechanically gated ion channels in sensory neurons are poorly understood. We show that FM1-43, a styryl dye used to fluorescently label cell membranes, permeates mechanosensitive ion channels in cultured dorsal root ganglion neurons, resulting in blockade of three previously defined subtypes of mechanically activated currents. Blockade and dye uptake is voltage dependent and regulated by external Ca^2+^. The structurally related larger dye FM3-25 inhibited mechanically activated currents to a lesser degree and did not permeate the channels. *In vivo*, FMI-43 decreases pain sensitivity in the Randall-Selitto test and increases the withdrawal threshold from von Frey hairs, together suggesting that the channels expressed at the cell body in culture mediate mechanosensation in the intact animal. These data give further insight into the mechanosensitive ion channels expressed by somatosensory neurons and suggest FM dyes are an interesting tool for studying them.

## Background

FM1-43 is a cationic styryl pyridinium dye used to fluorescently label biological membranes. It is used to study endocytosis, exocytosis and endosome trafficking and, in particular, synaptic vesicle recycling [1,2]. Gale *et al *[3] recently showed that FM1-43 acts as a permeant blocker of mechanotransducing ion channels in murine hair cells. FM1-43 applied to the extracellular surface of hair cells was taken up by the cell (resulting in fluorescent labelling of the cytoplasm) at a site close to the proposed site of transduction in a manner dependent on mechanical stimulation. FM1-43 was also shown to inhibit mechanically activated currents in a voltage-dependent manner where the lowest IC_50 _was 1.2 μM at -4 mV. Meyers *et al *[4] also reported that FM1-43 is a permeant blocker of hair cell mechanosensitive channels and showed that the dye permeated the non-selective cation channels TRPV1 and P2X_2_. When AM1-43, a fixable analogue of FM1-43, was injected subcutaneously, after 24–48 hours labelling of a number of sensory organs was observed. Interestingly, most of these structures had mechanosensory functions; they included hair cells in the cochlea and vestibular organs, Merkel cells and their neurites, muscle spindles, corneal nociceptors and enteric neurons. Fluorescence also accumulated (to varying degrees) in the cell bodies of all sensory neurons of the dorsal root ganglia (DRG) and other sensory ganglia [4]. In experiments using local dye injections and nerve ligation it was shown that dye entry into sensory neurons was via uptake at their peripheral, sensory terminals [4].

Mechanically activated (MA) cationic currents in cultured DRG display properties that correspond to their *in vivo *physiological function [5-7]. In this study the effects of FM1-43 on MA currents in cultured sensory neurons and on behavioural responses to noxious mechanical stimuli were investigated. The effects of the related dye FM3-25, which has two 18 carbon chains, rather than 2 butyryl groups on either side of the polar head, were also analysed in these two systems. The results show that FM1-43 is a permeant blocker of DRG neuron mechanosensitive ion channels, whereas FM3-25 is a non-permeant antagonist of these channels. In behavioural tests, these dyes inhibited responses in two assays of mechanosensitivity.

## Materials and methods

### Cell culture

All reagents from Sigma unless stated otherwise. Neonatal (P1) Sprague Dawley rats were decapitated, and 25–30 DRG were taken from each and digested in a solution of collagenase (Type XI, 0.6 mg/ml), dispase (3 mg/ml) and glucose (1.8 mg/ml) in Ca^2+^/Mg^2+ ^free PBS (Gibco) for 25 minutes prior to mechanical trituration. Cells were cultured in Dulbecco's modified Eagle medium containing 10% foetal bovine serum (Gibco), 2 mM glutamine (Gibco), 10,000 IU/ml penicillin/streptomycin (Gibco) and 100 ng/ml nerve growth factor on poly-L-lysine- and laminin-coated dishes and used the day after preparation.

### Electrophysiology

Whole-cell, perforated patch recordings were made using an Axopatch 200B amplifier (Axon Instruments) controlled by pCLAMP 9 (Axon Instruments). Patch pipettes were made from thin-walled glass (Harvard Apparatus) and had an initial resistance of 2–3 MΩ when filled with internal solution. Seals had a series resistance of 4–10 MΩ compensated for by 40–60% (feedback lag; 18 μs). Voltage-clamp recordings were made at a holding potential of -70 mV unless otherwise stated.

The standard intracellular solution contained (in mM): 110methanesulfonic acid, 30 KCl, 1 MgCl_2 _and 10 HEPES, pH 7.35(pH was corrected using KOH; final K^+ ^concentration ≈140 mM); 200 μg/ml amphotericin B was added immediately before recording. When testing the blocking efficiency of FM1-43 on inward and outward currents a caesium based internal solution was used (in mM): 110 caesium methane sulfonate, 30 CsCl, 1 MgCl_2_, 10 HEPES, pH 7.3 (adjusted using CsOH). The standard external solution contained (in mM): 140 NaCl, 4 KCl, 2 CaCl_2_, 1 MgCl_2 _and 10HEPES, pH 7.4 (adjusted with NaOH). Low pH solutions were made with 10 mM morpholinoethansulfonic acid in place of HEPES. FM1-43 (5/10 mM), FM3-25 (3 mM) and capsaicin (10 mM) stock solutions were made in DMSO.

### Mechanical stimulation and drug application

A heat-polished glass pipette was used to mechanically stimulate neurons [5,6]. The probe was controlled by a piezo-electric crystal drive (Burleigh) (tip diameter ≈5 μm) and was positioned at an angle of 70° to the surface of the culture dish. The probe was moved at a speed of 0.5 μm/msec and the stimulus was applied for 200 msec (unless stated otherwise). Cells that showed a reproducible response to mechanical stimuli (>200 pA stimulated at 20 sec intervals) were selected for further experimentation. Action potentials were recorded in the current-clamp configuration and were evoked by 1 msec square waves of depolarising current. Capsaicin (1 μM) and low pH (pH 5.3) were applied for 4 sec using a multi-barrel rapid solution changer (Biologic). Experiments were done at room temperature.

FM1-43 was visualised by excitation at 479 nm and through FITC filters. Images were acquired using Openlab software and further analysed using MCID Basic software. Samples of pixel intensity were taken from 5 random areas within the neuronal cytoplasm and 6 from the background region adjacent to the cells and the mean of the latter was subtracted from that of the former.

### Behavioural testing

For all experiments, male, 6–8 week, 20–25 g mice were used and the experimenter was blind to the treatment given to each animal. Withdrawal thresholds to punctate mechanical stimuli were tested using von Frey hairs applied to the plantar surface of the left hind paw. Mice were habituated to the testing conditions for 1.5 hours. Then, following acquisition of control data, intraplantar injections of drug or vehicle (20 μl, 5% DMSO in standard external solution, see above) were given and withdrawal thresholds were retested 20–40 minutes post-injection. 50% withdrawal thresholds were calculated using the "up-down" method [8,9].

For experiments using the Randall-Sellito device, mice were placed in a restraining tube and pressure was applied to a point midway along the tail. An ascending pressure ramp was applied until the animal showed obvious signs of discomfort and this pressure was taken as the pain threshold. Three control recordings were taken prior to injection of vehicle or drug (80 μl was given on the dorsal and ventral side of the tail) and then 4 further measurements were made. The first control and first post injection tests were discarded from analysis, to control for effects of learning and stress, respectively.

Data were analysed using Sigmaplot 8 and Sigmastat 4 software. Data are presented as mean ± standard error (unless otherwise stated).

## Results

**Figure 1:**
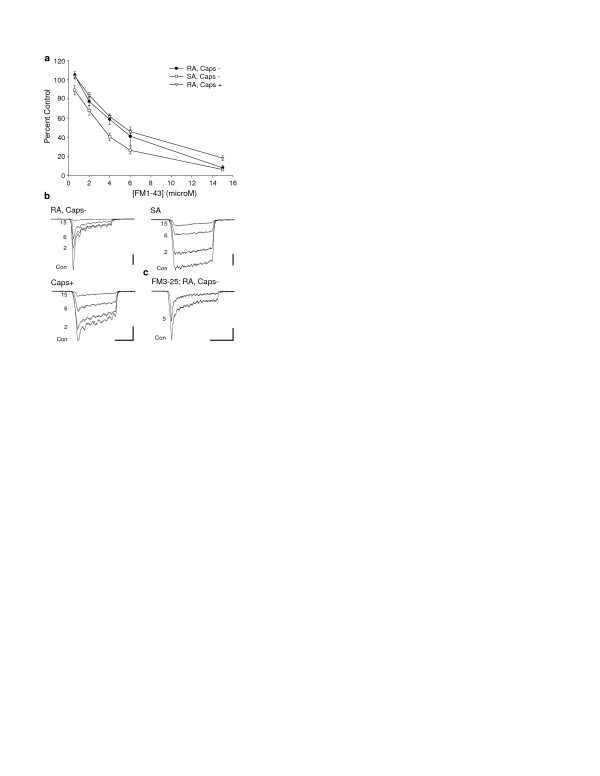
**Inhibition of MA currents by the styryl dyes FM1-43 and FM3-25**. (A) Concentration-inhibition functions for FM1-43 for inhibition of 3 classes of MA currents (each data point, n = 3–10). FM1-43 inhibited SA currents (Caps- neurons) at lower concentrations than RA currents in either Caps- or Caps+ neurons (2-way ANOVA, *P *< 0.001). At 0.6 μM FM1-43 had a slight facilitatory effect on currents. (B) Example traces showing inhibition of 3 classes of MA currents by 2, 6 and 15 μM FM1-43. (C) Inhibition of a RA current (Caps- neuron) by 5 μM FM3-25. All vertical scale bars: 0.2 nA. Horizontal scale bars: (B) 50 msec, (C) 100 msec.

To determine whether FM1-43 inhibited mechanically activated (MA) currents in DRG neurons, currents were recorded in the presence of extracellular FM1-43 at a range of concentrations from 0.6 to 15.0 μM. Cultured neurons generate MA currents that vary in magnitude and kinetics according to neuronal subtype. A subpopulation of nociceptive neurons generates slowly adapting currents (<25% adaptation) whereas in cultures from neonatal animals, most currents show mainly rapid adaptation (RA); RA currents in capsaicin insensitive (Caps-) are typically faster than in capsaicin sensitive (Caps +) neurons but the distinction is not as clear as in adult neurons [[5,6] manuscript submitted]. In this study, FM1-43 blocked rapidly- and slowly-adapting MA currents in both Caps- and Caps+ neurons. The effect was relatively similar on all three classes of currents (Figs. 1a,b) although overall the inhibitory effect was greater on SA currents than either RA population (2-way ANOVA, *P *< 0.001, Figs. 1a,b). From the concentration-inhibition plots it can be seen that 50% inhibition is achieved at around 3 μM for SA currents and around 5 μM for RA currents in Caps- and Caps+ neurons (Fig. 1a). The related dye FM3-25 was also tested; in contrast to the inactivity displayed by the compound at hair cell mechanotransduction channels, 5 μM FM3-25 inhibited DRG MA currents by 30.3 ± 4.2% (n = 9, Fig. 1c).

**Figure 2:**
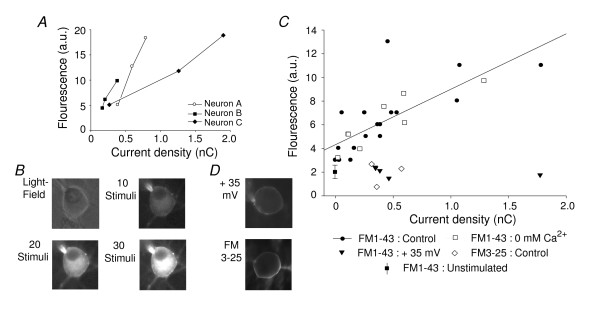
**Cytoplasmic accumulation of FM1-43 via permeation through MS ion channels**. Using the perforated patch configuration allowed dye accumulation in the cytoplasm to be measured. (A) Cytoplasmic fluorescence through FM1-43 uptake increased in 3 neurons after 10, 20 and 30 mechanical stimuli; shown is Intensity of fluorescent labelling against cumulative charge transfer. (B) Example images of a neuron (light field image, *top left*) following 10, 20 and 30 mechanical stimuli. N.B. the dye does not enter the nucleus. (C) Accumulation of FM1-43 is dependent on MS channel activity. In control conditions (standard external solution, membrane potential; -70 mV, ● solid line) fluorescent intensity is correlated with the amount of channel activity (as total charge transfer)(n = 18, Spearman's ranked order, *r *= 0.83, *P *< 0.001, fit: solid line). Removal of external Ca^2+ ^(□) had no apparent effect on dye uptake (n = 8, Spearman's ranked order, *r *= 0.83, *P *< 0.001, fit dotted line, partly occluded). Neither application of FM1-43 when the neuron was held at +35 mV (n = 5, ▼) nor application of FM3-25 (at -70 mV, n = 3, ◇) resulted in significant cytoplasmic fluorescence. Also shown is average background labelling after FM1-43 exposure in the absence of mechanical stimulation (n = 10, standard deviation indicated, ■). *D*. Examples of neurons stimulated in FM1-43 at +35 mV (*top*) and in FM3-25 at -70 mV (*bottom*).

Permeation of FM1-43 through ion channels results in fluorescent labelling of the cytoplasm [3,4]. Therefore we compared cytoplasmic dye accumulation to the total amount of MS channel activity evoked in the presence of FM1-43 or FM3-25. (This was enabled by the use of the perforated patch technique, as the dye is unable to pass through the pores formed by amphotericin B.) When mechanical stimuli were applied in the presence of 5 μM FM1-43, cytoplasmic labelling was correlated with channel activity. Three neurons were mechanically stimulated 30 times (at 9 μm) and after the 10^th^, 20^th ^and 30^th ^stimuli cytoplasmic florescence was measured; in each cell fluorescence was seen to increase with the number of stimuli (Figs. 2a,2b). Moreover, comparison of 18 neurons (with widely varying MA current amplitudes) showed that the intensity of fluorescent labelling after mechanical stimulation (10 × 9 μm) was strongly correlated to the total charge transfer (*r *= 0.82, *P *= 0.002, Pearson's product moment, Fig. 2c). FM1-43 can be internalised through endocytosis [1,2] so Ca^2+ ^influx through mechanogated ion channels could induce vesicular trafficking in neurons proportional to the extent of channel activity. To exclude this possibility, it was shown that cytoplasmic loading was indistinguishable from that in control conditions when MA currents were evoked in FM1-43 in Ca^2+ ^free extracellular solution (Fig. 2c). Consistently, when 3 neurons were mechanically stimulated in the presence of 5 μM FM3-25 (which also binds cell membranes) no significant labelling of the neuronal cytoplasm was observed (Figs. 2c,d), suggesting that the molecule cannot permeate the channel and that FM1-43 uptake is not via non-specific membrane binding. Finally, FM1-43 uptake did not occur when mechanically stimulated neurons were held at +35 mV showing that dye influx is voltage-dependent (Figs. 2c,d).

**Figure 3:**
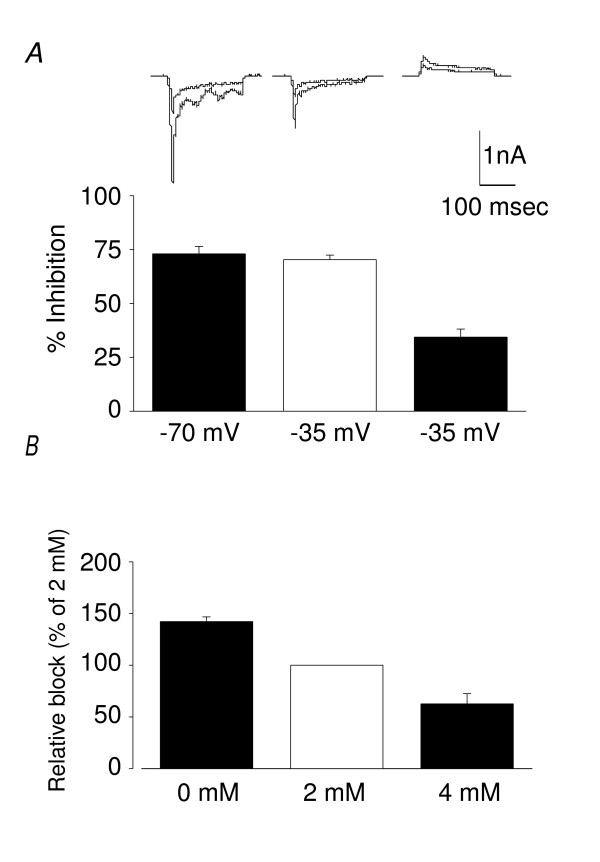
**Membrane potential and external Ca^2+ ^concentration affect inhibitory actions of FM1-43**. (A) Inhibition of MA currents at -70, -35 and +35 mV holding potentials (Caps- neurons, RA currents, n = 7). Activity was indistinguishable at -70 mV (69.7 ± 4.3% inhibition) and -35 mV (70.3 ± 2.1%) but was significantly lower at +35 mV (34.1 ± 2.1%, Student's paired *t*-test, *P *< 0.01). *Top*, example traces from the same cell. (B) Effect of Ca^2+ ^on FM1-43 activity. Increasing external Ca^2+ ^from 2 to 4 mM decreased the inhibitory activity of FM1-43 by 37.4 ± 9.8% (n = 3, Student's paired *t*-test, *P *< 0.05) whereas removing external Ca^2+ ^(no chelator included) increased the effect of FM1-43 to 142.2 ± 4.5% of control levels (n = 6, Student's paired *t*-test, *P *< 0.05).

The voltage dependence of current inhibition by permeant blockers is complex; as the membrane potential becomes more negative there is greater binding of the compound to the channel pore (increases blockade) but also greater passage through the channel (decreases blockade) (see Ref. 3). The ability of FM1-43 to inhibit MA currents was assessed at 3 membrane potentials (-70, -35 and +35 mV). In these experiments FM1-43 was an equally efficient blocker of MA currents at -70 and -35 mV (69.7 ± 4.3% versus 70.3 ± 2.1% inhibition, n = 4–5) but its blocking activity was significantly reduced at the positive holding potential (34.1 ± 2.1%, n = 7, *P *= 0.01, Fig. 3a). This is again consistent with it permeating and blocking the underlying ion channel. Both Ca^2+ ^[10] and FM1-43 act as permeant blockers of auditory mechanotransduction ion channels [3,4] and Ca^2+ ^is also a permeant blocker of DRG mechanosensitive ion channels [5,11]. Therefore, to determine if the two molecules interact, the inhibitory effect of FM1-43 on currents was tested when the Ca^2+ ^concentration was either reduced to nominally zero or doubled to 4 mM. Raising Ca^2+ ^levels to 4 mM inhibited MA currents by 32.0 ± 1.1% whilst inhibition of MA currents by 5 μM FM1-43 fell from 62.4 ± 0.6% to 39.1 ± 6.1% (n = 3, Caps-, RA currents, P < 0.05, Fig. 3b). Conversely, removing external Ca^2+ ^(no chelator included) increased the effect of FM1-43 to 142.2 ± 4.5% of control levels (n = 3, Student's paired *t*-test, *P *< 0.05).

**Figure 4:**
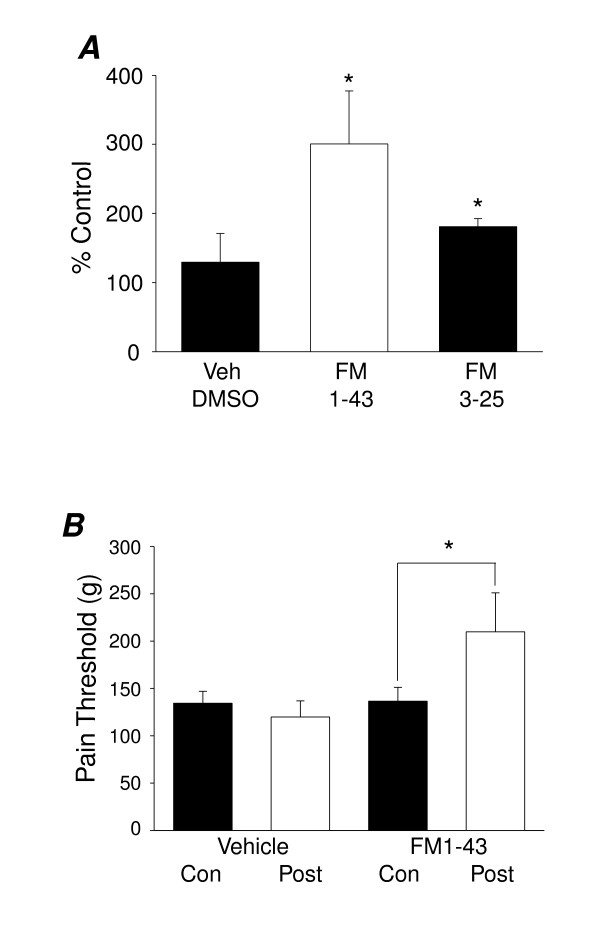
**FM1-43 inhibits behavioural responses to noxious mechanical stimuli**. (A) 5 nMoles FM1-43 (*P *< 0.05, n = 7, average increase above pre-injection control 200.7 ± 76.6%) and FM3-25 (*P *< 0.05, n = 3, 80.9 ± 11.6%) both increased the 50% withdrawal threshold in the von Frey test. Injection of the vehicle had no significant effect. (B) Using the mouse tail to test FM1-43 in the Randall-Selitto assay it was found that FM1-43 increased pain thresholds by 55.2 ± 23.9 % (2 × 20 nMoles top and bottom of tail injected, n = 6, Student's unpaired t-test versus vehicle effect, *P *< 0.05) whilst vehicle injection had no significant effect on thresholds (n = 6). Injection of the 5% DMSO vehicle (n = 9) had no significant effect.

We have proposed that the mechanosensitive ion channels expressed on the cell bodies of cultured neurons are normally present on the sensory nerve terminal *in vivo *where they mediate mechanosensation. To test this, we first examined whether peripheral application of FM1-43 to the stimulated region of mouse paws affected mechanically evoked paw withdrawal behaviour. Using von Frey hairs to assess withdrawal threshold in response to punctate mechanical stimuli, intraplantar injection of FM1-43 (5 nMoles) led to an increase in the 50% paw withdrawal threshold of over 100% (1.3 ± 0.2 g to 2.9 ± 0.4 g, *P *< 0.05, n = 7 Fig. 4a). Interestingly, in 3 animals tested in using von Frey hairs, intraplantar FM3-25 (5 nMoles) increased the 50% withdrawal threshold by approximately 75% (1.1 ± 0.3 g to 1.9 ± 0.5 g, n = 3, *P *< 0.05, Fig. 4a). Secondly, we also found FM1-43 to have an inhibitory action in the Randall-Selitto test, in which ascending pressure levels are applied to the tail until a pain-related behaviour is evoked. Here, local injection of 40 nMoles FM1-43 into the tail increased pain thresholds by approximately 50% (effect significant relative to vehicle effect *P *< 0.05, n = 6, Fig 4b).

FM1-43 does not block acid sensing ion channel (ASIC) mediated currents in DRG neurons; at 20 μM FM1-43 slightly potentiated transient proton-gated currents from 3.81 ± 0.22 nA to 4.10 ± 0.32 nA (107.0 ± 3.1%, n = 5, *P *< 0.05). When cells were exposed to low pH (pH5.4) in the presence of 5 μM FM1-43 staining was observed in some neurons and not others (0.077 ± 0.019 arbitrary intensity units, n = 5) but there was no correlation between labelling and total charge transfer (*r *= 0.394, *P *= 0.512, data not shown). In 4 cells action potentials were elicited in the presence of 5 μM FM1-43; cytoplasmic labelling was not significantly above background after 50 action potentials (0.026 ± 0.007 vs 0.020 ± 0.002). This concentration of FM1-43 caused a slight depolarisation of the membrane potential (-57.5 to -55.8 mV, n = 6, *P *< 0.05), but did not affect action potential threshold, duration, amplitude or the maximal rate of depolarisation (data not shown).

## Discussion

We have demonstrated that FM1-43 acts as a permeant blocker of mechanosensitive ion channels expressed by cultured DRG neurons and that when given peripherally it inhibits behavioural responses to mechanical stimuli. It had previously been shown that FM1-43 blocks and permeates mechanosensitive ion channels of hair cells [3,4]; although these two channel types have a number of distinct properties [5] the action of FM1-43 was similar. Both channels were blocked by low micromolar concentrations of the drug and in both cases fluorescent labelling of the cytoplasm was induced by mechanical stimulation. In this study, using the perforated patch technique, the degree of staining was found to correlate with the amount of mechanosensitive ion channel activity. The efficacy of FM1-43 in inhibiting slowly adapting currents was slightly but significantly higher than that for rapidly adapting currents, suggesting that distinct ion channels may be responsible for the two currents.

Calcium is also a permeant blocker of hair cell channels [10,11] and mechanosensitive ion channels in DRG neurons [5,12]. Here increasing external calcium levels reduced the potency with which FM1-43 blocked MA currents. Hence, the data in the two auditory papers and this work are consistent with the divalent cation FM1-43 interacting with the channel pore in a manner similar to calcium. Permeation of FM1-43 is further supported by the observation that its inhibitory action was not reduced by depolarising the neuron to -35 mV, but it was reduced by holding it at +35 mV. For permeant blockers, the voltage dependence is determined by the membrane potential drawing the compound into the channel pore and subsequently encouraging expulsion into the cytoplasm; thus at more negative potentials the compound enters the channel more readily but also passes through it more rapidly (See Ref. 3). At +35 mV the dye did not significantly enter the cell following mechanical stimulation suggesting that at this membrane potential there was an insufficient electrochemical gradient for FM1-43 to permeate the channel.

FM3-25 inhibited MA currents in DRG neurons by around 30% at 5 μM, in contrast to auditory transduction channels where it is inactive (up to 30 μM) [3]. However, as in hair cells, FM3-25 was not taken up by the cell in response to mechanical stimulation. These data suggest that the polar head group of FM3-25 is able to block the DRG channel but the larger size of FM3-25 makes it impermeant.

Local application of FM1-43 and FM3-25 blocked behavioural responses to noxious mechanical stimuli as assessed by von Frey hairs and the Randall-Selitto device. Both compounds increased paw withdrawal thresholds in response to punctate stimulation with von Frey hairs and it is notable that the degree of behavioural inhibition appeared directly related to the compounds' efficiency in blocking MA currents. Whereas this test measures the acute withdrawal from a punctate stimulus the Randall-Selitto device measures a response to high levels of pressure related to C-fibre activation. FM1-43 did not affect action potential generation so the effect of the dye is likely dependent on inhibition of transduction. This is the first demonstration of a mechanosensitive ion channel antagonist inhibiting a mammalian behavioural response to mechanical stimulation. The results are consistent with the ion channels we have characterised on the somata of cultured neurons being expressed at the peripheral terminals of DRG fibres where they mediate sensory mechanotransduction.

Following systemic administration of the FM1-43 analogue AM1-43 primary receptor cells in a number of mechanosensory systems were labelled with this dye [4]. Given the absence of cell labelling following action potential trains, we speculate that permeation through mechanosensitive ion channels is a major entry point for styryl dyes into sensory neurons when they are given systemically. In the auditory system FM1-43 has become an important tool in the study of mechanotransduction; it has been used in knockdown studies [13,14] and for investigation of the maturation of this system [15,16]. This study has characterised the action of FM1-43 on DRG mechanosensitive ion channels, demonstrated that this compound can inhibit behavioural responses and suggests that this dye is a useful tool for the study of somatosensory mechanotransduction.
